# Scheelite-type sodium neodymium(III) *ortho*-oxidomolybdate(VI), NaNd[MoO_4_]_2_

**DOI:** 10.1107/S1600536811046976

**Published:** 2011-11-09

**Authors:** Thomas Schleid, Ingo Hartenbach

**Affiliations:** a Institut für Anorganische Chemie, Universität Stuttgart, Pfaffenwaldring 55, 70569 Stuttgart, Germany

## Abstract

Scheelite-type NaNd[MoO_4_]_2_ contains one crystallographic position (site symmetry 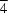) for the large cations, which is mixed-occupied by Na^+^ and Nd^3+^ cations in a 1:1 molar ratio. Thus, both are surrounded by eight O atoms in the shape of a trigonal dodeca­hedron. Furthermore, the structure consists of crystallographically unique [MoO_4_]^2−^ units (site symmetry 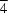) surrounded by eight sodium and neodymium cations, which are all vertex-attached. The polyhedra around the Na^+^/Nd^3+^ cations are connected to four others *via* common edges, building up a three-dimensional network in whose tetra­hedral voids of O atoms the Mo^6+^ cations reside.

## Related literature

For isotypic Na*Ln*[MoO_4_]_2_ structures, see: Stevens *et al.* (1991 ▶) and Teller (1992 ▶) for *Ln* = La; Teller (1992 ▶) for *Ln* = Ce; Zhao *et al.* (2010 ▶) for *Ln* = Er. For inter­penetrating diamond-like networks, see: Schustereit *et al.* (2011 ▶). These were also obseverd in NaTl, see: Zintl & Dullenkopf (1932 ▶).

## Experimental

### 

#### Crystal data

NaNd[MoO_4_]_2_
*M* *_r_* = 487.11
Tetragonal, 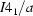

*a* = 5.2871 (3) Å
*c* = 11.5729 (7) Å
*V* = 323.50 (3) Å^3^
*Z* = 2
Mo *K*α radiation
μ = 11.79 mm^−1^
*T* = 293 K
0.11 × 0.09 × 0.07 mm

#### Data collection

Nonius KappaCCD diffractometer
Absorption correction: numerical (*X-SHAPE*; Stoe & Cie, 1995 ▶) *T*_min_ = 0.273, *T*_max_ = 0.434
1026 measured reflections
196 independent reflections
135 reflections with *I* > 2σ(*I*)
*R*_int_ = 0.058

#### Refinement

*R*[*F* ^2^ > 2σ(*F* ^2^)] = 0.020
*wR*(*F* ^2^) = 0.044
*S* = 0.99
196 reflections
15 parameters
Δρ_max_ = 0.45 e Å^−3^
Δρ_min_ = −0.42 e Å^−3^

### 

Data collection: *COLLECT* (Nonius, 1998 ▶); cell refinement: *SCALEPACK* (Otwinowski & Minor, 1997 ▶); data reduction: *SCALEPACK* and *DENZO* (Otwinowski & Minor, 1997 ▶); program(s) used to solve structure: *SHELXS97* (Sheldrick, 2008 ▶); program(s) used to refine structure: *SHELXL97* (Sheldrick, 2008 ▶); molecular graphics: *DIAMOND* (Brandenburg, 2006 ▶); software used to prepare material for publication: *SHELXL97*.

## Supplementary Material

Crystal structure: contains datablock(s) I, global. DOI: 10.1107/S1600536811046976/hp2019sup1.cif

Structure factors: contains datablock(s) I. DOI: 10.1107/S1600536811046976/hp2019Isup2.hkl

Additional supplementary materials:  crystallographic information; 3D view; checkCIF report

## Figures and Tables

**Fig. 1.**
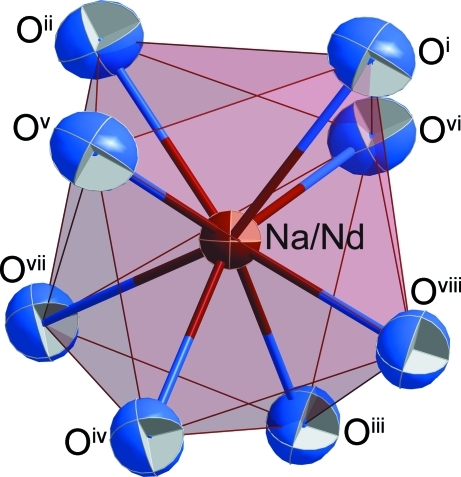
Trigonal dodecahedral oxygen environment of the Na+/Nd3+ cations in Scheelite-type NaNd[MoO4]2 (ellipsoids are drawn at 90 % probability level, symmetry codes: (i) y–1/4, –x+3/4, z+3/4;(ii) –y+1/4, x–1/4, z+3/4; (iii) x–1/2, y, –z+1/2; (iv) –x+1/2, –y+1/2, –z+1/2; (v) –y+3/4, x–1/4, –z+3/4; (vi) y–3/4, –x+3/4, –z+3/4; (vii) x–1/2, y–1/2, z+1/2; (viii) –x+1/2, –y+1, z+1/2)

**Fig. 2.**
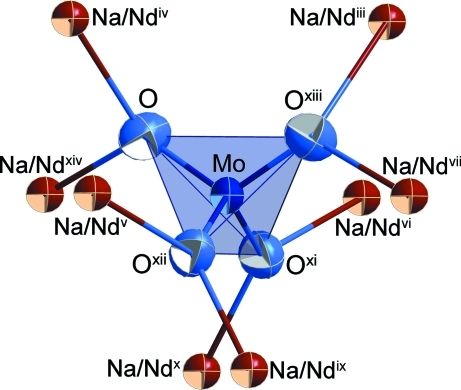
Cationic surrounding of the isolated ortho-oxomolybdate(VI) tetrahedra [MoO4]2– in Scheelite-type NaNd[MoO4]2 (ellipsoids are drawn at 90 % probability level, symmetry codes for O: x, y, z; (xi) –y+1/4, x+1/4, –z+1/4; (xii) y–1/4, –x+1/4, z+1/4; (xiii) –x, –y+1/2, z; symmetry codes for Na/Nd: (iii) x–1/2, y, –z+1/2; (iv) –x+1/2, –y+1/2, –z+1/2; (v) –y+3/4, x–1/4, –z+3/4; (vi) y–3/4, –x+3/4, –z+3/4; (vii) x–1/2, y–1/2, z+1/2; (ix) –x, –y, –z+1; (x) –x, –y+1, –z+1; (xiv) x+1/2, y+1/2, z–1/2)

**Fig. 3.**
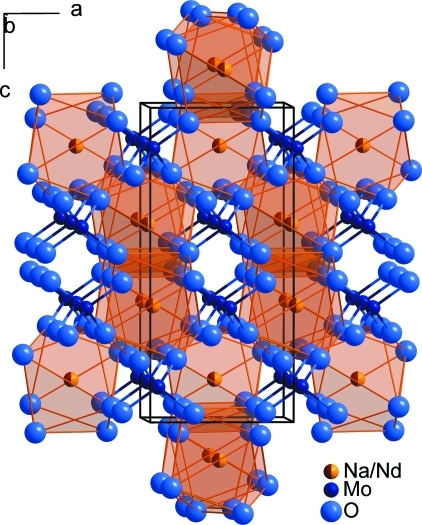
View at the crystal structure of Scheelite-type NaNd[MoO4]2 along [010] (slightly rotated) with special emphasis on the edge-connected oxygen polyhedra around the Na+ and Nd3+ cations, respectively.

## supplementary crystallographic information

### Comment

Scheelite-type sodium lanthanide *ortho*-oxomolybdates of the formula Na*Ln*[MoO_4_]_2_ are already known for *Ln* = La, Ce, and Er (see related literature). The structure features Na^+^ and Nd^3+^ cations together at the common *Wyckoff* position 4*b*, eightfold coordinated by O^2–^ anions in the shape of trigonal dodecahedra (Fig. 1). The Na : Nd ratio was fixed at a molar ratio of 1 : 1 for maintaining electroneutrality. A similar surrounding is found for the cationic coordination around the crystallographically unique isolated *ortho*-oxomolybdate anions [MoO_4_]^2–^ with the Mo^6+^ cations at the *Wyckoff* position 4*a*  (Fig. 2). The polyhedra around the Na^+^/Nd^3+^ cations are interconnected to four others via common edges building up a three-dimensional network, in whose voids of oxygen the Mo^6+^ cations are located (Fig. 3). Both the cations at the sites 4*a* (Na^+^ and Nd^3+^ in a 1:1 molar ratio) and 4*b* (Mo^6+^) arrange themselves in two interpenetrating *diamond*-like networks (Schustereit *et al.*, 2011) as in the case of NaTl (Zintl & Dullenkopf, 1932).

### Experimental

Pale violet, coarse single crystals of Scheelite-type NaNd[MoO_4_]_2_ were obtained as by-product in an unsuccessful attempt to synthesize NdF[MoO_4_], using a mixture of NdF_3_ and Na_2_[MoO_4_] in a 1:1 molar ratio, which was heated at 850 °C for 7 days in an evacuated, sealed, fused-silica ampoule.

### Crystal data

**Table:**

NaNd[MoO_4_]_2_	*D*_x_ = 5.001 Mg m^−^^3^
*M**_r_* = 487.11	Mo *K*α radiation, λ = 0.71069 Å
Tetragonal, *I*4_1_/*a*	Cell parameters from 2325 reflections
Hall symbol: -I 4ad	θ = 1.0–28.3°
*a* = 5.2871 (3) Å	µ = 11.79 mm^−^^1^
*c* = 11.5729 (7) Å	*T* = 293 K
*V* = 323.50 (3) Å^3^	Coarse transparent, pale violet
*Z* = 2	0.11 × 0.09 × 0.07 mm
*F*(000) = 438	

### Data collection

**Table:**

Nonius KappaCCD diffractometer	196 independent reflections
Radiation source: fine-focus sealed tube	135 reflections with *I* > 2σ(*I*)
graphite	*R*_int_ = 0.058
ω and φ scans	θ_max_ = 28.2°, θ_min_ = 4.2°
Absorption correction: numerical (*X-SHAPE*; Stoe & Cie, 1995)	*h* = −6→6
*T*_min_ = 0.273, *T*_max_ = 0.434	*k* = −6→6
1026 measured reflections	*l* = −15→15

### Refinement

**Table:**

Refinement on *F*^2^	Primary atom site location: structure-invariant direct methods
Least-squares matrix: full	Secondary atom site location: difference Fourier map
*R*[*F*^2^ > 2σ(*F*^2^)] = 0.020	*w* = 1/[σ^2^(*F*_o_^2^) + (0.0102*P*)^2^] where *P* = (*F*_o_^2^ + 2*F*_c_^2^)/3
*wR*(*F*^2^) = 0.044	(Δ/σ)_max_ < 0.001
*S* = 0.99	Δρ_max_ = 0.45 e Å^−^^3^
196 reflections	Δρ_min_ = −0.42 e Å^−^^3^
15 parameters	Extinction correction: *SHELXL97* (Sheldrick, 2008), Fc^*^=kFc[1+0.001xFc^2^λ^3^/sin(2θ)]^-1/4^
0 restraints	Extinction coefficient: 0.0092 (8)

### Special details

**Table:**

Geometry. All esds (except the esd in the dihedral angle between two l.s. planes) are estimated using the full covariance matrix. The cell esds are taken into account individually in the estimation of esds in distances, angles and torsion angles; correlations between esds in cell parameters are only used when they are defined by crystal symmetry. An approximate (isotropic) treatment of cell esds is used for estimating esds involving l.s. planes.
Refinement. Refinement of F^2^ against ALL reflections. The weighted R-factor wR and goodness of fit S are based on F^2^, conventional R-factors R are based on F, with F set to zero for negative F^2^. The threshold expression of F^2^ > 2sigma(F^2^) is used only for calculating R-factors(gt) etc. and is not relevant to the choice of reflections for refinement. R-factors based on F^2^ are statistically about twice as large as those based on F, and R- factors based on ALL data will be even larger.

### Fractional atomic coordinates and isotropic or equivalent isotropic displacement parameters (Å^2^)

**Table:**

	*x*	*y*	*z*	*U*_iso_*/*U*_eq_	Occ. (<1)
Na	0.0000	0.2500	0.6250	0.0125 (2)	0.50
Nd	0.0000	0.2500	0.6250	0.0125 (2)	0.50
Mo	0.0000	0.2500	0.1250	0.0131 (2)	
O	0.2406 (3)	0.3949 (3)	0.04140 (14)	0.0255 (5)	

### Atomic displacement parameters (Å^2^)

**Table:**

	*U*^11^	*U*^22^	*U*^33^	*U*^12^	*U*^13^	*U*^23^
Na	0.0130 (3)	0.0130 (3)	0.0113 (3)	0.000	0.000	0.000
Nd	0.0130 (3)	0.0130 (3)	0.0113 (3)	0.000	0.000	0.000
Mo	0.0122 (3)	0.0122 (3)	0.0151 (3)	0.000	0.000	0.000
O	0.0298 (10)	0.0227 (12)	0.0239 (9)	−0.0002 (8)	0.0022 (8)	−0.0008 (9)

### Geometric parameters (Å, °)

**Table:**

Na—O^i^	2.4851 (15)	Na—Nd^x^	3.91907 (16)
Na—O^ii^	2.4851 (15)	Na—Na^x^	3.91907 (16)
Na—O^iii^	2.4851 (15)	Mo—O^xi^	1.7725 (16)
Na—O^iv^	2.4851 (15)	Mo—O	1.7725 (17)
Na—O^v^	2.5182 (18)	Mo—O^xii^	1.7725 (16)
Na—O^vi^	2.5182 (18)	Mo—O^xiii^	1.7725 (16)
Na—O^vii^	2.5182 (18)	O—Nd^iii^	2.4851 (15)
Na—O^viii^	2.5182 (18)	O—Na^iii^	2.4851 (15)
Na—Na^ix^	3.91907 (17)	O—Na^xiv^	2.5182 (18)
Na—Nd^ix^	3.91907 (17)	O—Nd^xiv^	2.5182 (18)
			
O^i^—Na—O^ii^	126.90 (5)	O^vi^—Na—Nd^ix^	102.66 (4)
O^i^—Na—O^iii^	126.90 (5)	O^vii^—Na—Nd^ix^	85.20 (3)
O^ii^—Na—O^iii^	78.41 (7)	O^viii^—Na—Nd^ix^	130.62 (4)
O^i^—Na—O^iv^	78.41 (7)	Na^ix^—Na—Nd^ix^	0.0
O^ii^—Na—O^iv^	126.90 (5)	O^i^—Na—Nd^x^	38.74 (4)
O^iii^—Na—O^iv^	126.90 (5)	O^ii^—Na—Nd^x^	160.78 (4)
O^i^—Na—O^v^	151.28 (7)	O^iii^—Na—Nd^x^	101.53 (4)
O^ii^—Na—O^v^	68.38 (4)	O^iv^—Na—Nd^x^	68.65 (4)
O^iii^—Na—O^v^	76.88 (6)	O^v^—Na—Nd^x^	130.62 (4)
O^iv^—Na—O^v^	73.65 (3)	O^vi^—Na—Nd^x^	85.20 (3)
O^i^—Na—O^vi^	73.65 (3)	O^vii^—Na—Nd^x^	38.14 (3)
O^ii^—Na—O^vi^	76.88 (6)	O^viii^—Na—Nd^x^	102.66 (4)
O^iii^—Na—O^vi^	68.38 (4)	Na^ix^—Na—Nd^x^	123.025 (3)
O^iv^—Na—O^vi^	151.28 (7)	Nd^ix^—Na—Nd^x^	123.025 (3)
O^v^—Na—O^vi^	134.81 (8)	O^i^—Na—Na^x^	38.74 (4)
O^i^—Na—O^vii^	76.88 (6)	O^ii^—Na—Na^x^	160.78 (4)
O^ii^—Na—O^vii^	151.28 (7)	O^iii^—Na—Na^x^	101.53 (4)
O^iii^—Na—O^vii^	73.65 (3)	O^iv^—Na—Na^x^	68.65 (4)
O^iv^—Na—O^vii^	68.38 (4)	O^v^—Na—Na^x^	130.62 (4)
O^v^—Na—O^vii^	98.49 (3)	O^vi^—Na—Na^x^	85.20 (3)
O^vi^—Na—O^vii^	98.49 (3)	O^vii^—Na—Na^x^	38.14 (3)
O^i^—Na—O^viii^	68.38 (4)	O^viii^—Na—Na^x^	102.66 (4)
O^ii^—Na—O^viii^	73.65 (3)	Na^ix^—Na—Na^x^	123.025 (3)
O^iii^—Na—O^viii^	151.28 (7)	Nd^ix^—Na—Na^x^	123.025 (3)
O^iv^—Na—O^viii^	76.88 (6)	Nd^x^—Na—Na^x^	0.0
O^v^—Na—O^viii^	98.49 (3)	O^xi^—Mo—O	113.83 (11)
O^vi^—Na—O^viii^	98.49 (3)	O^xi^—Mo—O^xii^	107.34 (5)
O^vii^—Na—O^viii^	134.81 (8)	O—Mo—O^xii^	107.34 (5)
O^i^—Na—Na^ix^	160.78 (4)	O^xi^—Mo—O^xiii^	107.34 (5)
O^ii^—Na—Na^ix^	68.65 (4)	O—Mo—O^xiii^	107.34 (5)
O^iii^—Na—Na^ix^	38.74 (4)	O^xii^—Mo—O^xiii^	113.83 (11)
O^iv^—Na—Na^ix^	101.53 (4)	Mo—O—Nd^iii^	133.30 (9)
O^v^—Na—Na^ix^	38.14 (3)	Mo—O—Na^iii^	133.30 (9)
O^vi^—Na—Na^ix^	102.66 (4)	Nd^iii^—O—Na^iii^	0.0
O^vii^—Na—Na^ix^	85.20 (3)	Mo—O—Na^xiv^	120.23 (8)
O^viii^—Na—Na^ix^	130.62 (4)	Nd^iii^—O—Na^xiv^	103.12 (6)
O^i^—Na—Nd^ix^	160.78 (4)	Na^iii^—O—Na^xiv^	103.12 (6)
O^ii^—Na—Nd^ix^	68.65 (4)	Mo—O—Nd^xiv^	120.23 (8)
O^iii^—Na—Nd^ix^	38.74 (4)	Nd^iii^—O—Nd^xiv^	103.12 (6)
O^iv^—Na—Nd^ix^	101.53 (4)	Na^iii^—O—Nd^xiv^	103.12 (6)
O^v^—Na—Nd^ix^	38.14 (3)	Na^xiv^—O—Nd^xiv^	0.0

Symmetry codes: (i) *y*−1/4, −*x*+3/4, *z*+3/4; (ii) *x*−1/2, *y*, −*z*+1/2; (iii) −*x*+1/2, −*y*+1/2, −*z*+1/2; (iv) −*y*+1/4, *x*−1/4, *z*+3/4; (v) *x*−1/2, *y*−1/2, *z*+1/2; (vi) −*x*+1/2, −*y*+1, *z*+1/2; (vii) −*y*+3/4, *x*−1/4, −*z*+3/4; (viii) *y*−3/4, −*x*+3/4, −*z*+3/4; (ix) −*x*, −*y*, −*z*+1; (x) −*x*+1/2, −*y*+1/2, −*z*+3/2; (xi) −*x*, −*y*+1/2, *z*; (xii) −*y*+1/4, *x*+1/4, −*z*+1/4; (xiii) *y*−1/4, −*x*+1/4, −*z*+1/4; (xiv) *x*+1/2, *y*+1/2, *z*−1/2.
